# Effectiveness of social skills training conducted in a group of subjects with first-episode psychosis

**DOI:** 10.1192/j.eurpsy.2021.1352

**Published:** 2021-08-13

**Authors:** F. Brando, G.M. Giordano, P. Bucci, D. Palumbo, G. Piegari, A. Mucci, S. Galderisi

**Affiliations:** Department Of Psychiatry, University of Campania “Luigi Vanvitelli”, Naples, Italy

**Keywords:** social skills training, schizophrénia, psychiatric rehabilitation, first episode of psychosis

## Abstract

**Introduction:**

Cognitive deficits are considered a key feature of schizophrenia due to their substantial influence on the psychosocial outcome of subjects affected by this disorder. Several studies showed that moderate to severe cognitive impairments, including dysfunctions of social cognition, are already present during the early phases of the illness, in subjects with first-episode psychosis (FEPs). Psychosocial interventions, such as social skill training (SST), could therefore be implemented already upon occurrence of the first episode of psychosis to improve the overall functional outcome of schizophrenia, which represents to date an unmet need in the care of these patients.

**Objectives:**

The study aims to evaluate the use of SST to enhance social skills and real-life functioning in FEPs.

**Methods:**

The sample included 7 FEPs (age between 15 and 40). The SST intervention included 30 sessions lasting 2 hours and delivered twice a week. Psychopathology, neurocognition, real life functioning, functional capacity and social cognition were assessed at baseline ad after training. Paired samples t-tests were performed to evaluate the effects of the intervention. All subjects were treated with second generation antipsychotics.

**Results:**

Significant improvements were observed in negative symptoms, social cognition, problem solving skills, as well as in global functioning (all p<0.05). Within real-life functioning, the improvement was greater for the domain of interpersonal relationships.

**Conclusions:**

These preliminary findings suggest that SST might complement pharmacological treatment in FEPs to improve functional outcome in these subjects. Further studies with a higher sample size and a longer follow-up are required in order to confirm the present results.

